# Executive Functioning in Different Types of Reading Disabilities

**DOI:** 10.3390/jintelligence12100101

**Published:** 2024-10-14

**Authors:** Irene Cadime, Bruna Rodrigues, Iolanda Ribeiro, María Teresa Martín-Aragoneses

**Affiliations:** 1 Centro de Investigação em Estudos da Criança, Universidade do Minho, 4710-057 Braga, Portugal; 2 Centro de Investigação em Psicologia, Universidade do Minho, 4710-057 Braga, Portugal; bruna.fct.psi@gmail.com (B.R.); iolanda@psi.uminho.pt (I.R.); 3 Facultad de Educación, Universidad Nacional de Educación a Distancia (UNED), 28040 Madrid, Spain; mt.m.aragoneses@edu.uned.es; 4 Instituto Mixto de Investigación–Escuela Nacional de Sanidad (IMIENS), 28029 Madrid, Spain

**Keywords:** executive functions, reading disabilities, reading comprehension, fluency, rapid naming, working memory, cognitive flexibility, inhibition, processing speed

## Abstract

Students with reading disabilities form a heterogeneous group: some struggle with accurate and fast reading (dysfluent readers), others with comprehension (poor comprehenders), and some face challenges in both areas (poor readers). Research has indicated a link between executive functioning skills and reading performance; yet, further studies are necessary to fully understand the executive profiles in various types of reading disabilities. The goal of this study was to examine differences in executive functioning among three types of reading disabilities, comparing their performance with that of children without difficulties in either skill (typical readers). Ninety-one students from schools in Portugal participated in the study. The results reveal specific deficits in naming speed and cognitive flexibility in poor readers and dysfluent readers compared to the other groups. Additionally, poor readers exhibited significantly slower processing speed and lower working memory. However, no significant differences were observed in planning. Discriminant function analysis results indicated that the examined executive functions are better at discriminating groups with fluency deficits than those with comprehension difficulties. In conclusion, these results suggest distinct deficit patterns in executive functioning skills across different types of reading disabilities. Taking into account these findings is crucial for effective assessment and intervention with these children.

## 1. Introduction

Students with reading disabilities represent a heterogeneous group with different profiles of difficulties. In the literature, three types of students with reading disabilities are commonly identified: (i) those with decoding or fluency problems but adequate comprehension, usually referred to as poor decoders (this group also includes students with dyslexia) and dysfluent readers, respectively; (ii) those with comprehension problems but adequate decoding or fluency skills, commonly known as poor comprehenders (sometimes also referred to as children with specific reading comprehension difficulties), and (iii) those who present difficulties in both areas, frequently referred to as poor readers (Baron et al. 2019; Feng et al. 2023; Kelso et al. 2022). Research has also pointed out other subgroups within these types of reading disabilities. For example, Wolf and Bowers (1999) proposed a double-deficit hypothesis that was based on the idea that phonological deficits and the underlying processes that cause naming-speed deficits are two distinct factors contributing to developmental dyslexia. Subsequent research provided evidence for the existence of reader profiles who exhibited deficits only in phonological awareness, only in naming speed, or in both skills (Ozernov-Palchik et al. 2017), providing empirical support for Wolf and Bowers’ (1999) hypothesis. However, this study only included three groups of reading disabilities: (1) dysfluent readers; (2) poor comprehenders, and (3) poor readers.

Estimates across different countries suggest that poor decoders and students with dyslexia constitute around 5% to 10% of school-aged children (Al-Shidhani and Arora 2012; Feng et al. 2022; Vale et al. 2011; Wagner et al. 2020) and that 10% to 15% of the school-aged children experience specific comprehension difficulties (Leach et al. 2003; Rodrigues et al. 2023; Torppa et al. 2007). Therefore, when considering all types of reading disabilities, the prevalence may exceed 20% (Cecilia et al. 2014; U.S. Department of Education 2021; Torppa et al. 2007). Consequently, we decided not to restrict the present study to children with decoding deficits or dyslexia. We also included profiles with comprehension deficits to provide a more complete overview of the population of children with reading disabilities. We preferred to study children with oral reading fluency difficulties, rather than children with decoding difficulties alone, because the number of words read correctly per minute (which is an indicator of both accuracy and speed) has been shown to be a better indicator of children’s reading proficiency beyond the initial phase of learning to read, especially in languages with intermediate orthographic depth, such as European Portuguese (Cadime et al. 2017).

In the DSM5, reading disabilities are classified under the broader category of Specific Learning Disorders (SLDs), a neurodevelopmental disorder characterized by persistent and impairing difficulties in foundational learning skills due to deficits in the ability to process information efficiently and accurately (American Psychiatric Association 2013). Efficient and accurate information processing requires not only processing speed but also other cognitive skills, such as executive functions.

Executive functions are a set of cognitive processes essential for regulating behavior, managing time and attention, planning and executing goal-directed tasks, and problem-solving (Miyake et al. 2000; Starr et al. 2023). Traditionally, these functions have been conceptualized as comprising a triad of separate (but related) components: inhibition, cognitive flexibility, and updating (Diamond 2013; Miyake et al. 2000; Wu and Was 2023). Inhibition includes inhibitory and interference control, referring to the ability to suppress automatic or dominant responses, suppress competing stimuli or responses that are not relevant to the goal, and resist distractions from irrelevant stimuli (Daucourt et al. 2018; López-Vallejo et al. 2024). Cognitive flexibility, also known as shifting or switching, involves alternating attention between mental operations or tasks (Butterfuss and Kendeou 2018; Diamond 2013). Updating working memory entails tracking and evaluating incoming information, coding it, and replacing outdated information with the most current data (Miyake et al. 2000). The scientific literature also suggests that other higher-order functions, such as reasoning, problem solving, and planning, are built upon this triad of functions (Diamond 2013).

A recent model, the Active View of Reading (Duke and Cartwright 2021), built on the Simple View of Reading (Hoover and Gough 1990), posits that in addition to word recognition and language comprehension, reading comprehension is influenced by active self-regulation. Active self-regulation encompasses executive function skills, which the model identifies as a core contributor to successful reading comprehension and also as an indirect contributor via word recognition, as suggested by findings from previous studies (e.g., Cirino et al. 2019; Ober et al. 2019). This model also considers reading fluency as a bridging process between word recognition and language comprehension, which is consequently also influenced by executive functioning. However, the model does not specify whether specific executive functioning skills are differentially associated with each of the reading components.

### 1.1. Association Between Executive Functioning and Reading Comprehension Difficulties

Reading comprehension involves the construction of a mental model of a text (Kintsch and Rawson 2005). Classic reading comprehension models, such as the Construction–Integration model (Kintsch 1988) and the Structure Building Framework (Gernsbacher 1991), conceptualize how this mental model is built, often implicitly acknowledging the role of executive functions (for a review see Butterfuss and Kendeou 2018). Regardless of the model, working memory, inhibition, cognitive flexibility, and planning have all been hypothesized to play a role in reading comprehension.

Working memory is crucial to comprehension as it enables readers to hold in mind earlier information from the text, relate it to later content to maintain local coherence, and recall prior knowledge stored in the long-term memory, integrating it with the information from the text to generate inferences (Follmer 2018). Inhibition has also been hypothesized to play a role in reading comprehension, as it ensures that only task-relevant information accesses working memory, discards irrelevant stimuli, and suppresses dominant but inadequate responses to the situation, thereby mitigating the interference of distractions and outdated or irrelevant information during text comprehension (Borella et al. 2010; Butterfuss and Kendeou 2018). Cognitive flexibility is similarly believed to be involved in reading comprehension (Johann et al. 2020), as understanding a text requires the orchestration of multiple processes, including those involved in processing the phonological, morphosyntactic, and semantic features of the text while simultaneously engaging metacognitive processes (Butterfuss and Kendeou 2018).

Cognitive flexibility may also facilitate changing mental frameworks and forming new concepts by incorporating new and sometimes unexpected information into the text representation while reading is ongoing. It can support the inference-making process that often requires alternating between relevant pieces of information (Tabullo et al. 2023). Flexibility may also aid comprehension, as it is frequently necessary to switch between different reading strategies, such as making predictions and asking questions (Gnaedinger et al. 2016). Additionally, some authors have proposed a specific type of cognitive flexibility—the graphophonological-semantic cognitive flexibility—that is unique to reading. This type of flexibility “involves the ability to simultaneously consider and actively switch between the letter–sound (graphophonological) and meaning (semantic) features of printed words” (Duke and Cartwright 2021, p. 7). In the Active View of Reading (Duke and Cartwright 2021), graphophonological-semantic cognitive flexibility is understood as a bridging skill between word recognition and comprehension, and research has indeed found significant correlations between this skill and reading comprehension (Cartwright 2002; Cartwright et al. 2010, 2017; Gnaedinger et al. 2016).

Planning is another skill potentially associated with reading comprehension, as higher levels of comprehension are linked to the effective use of self-regulation skills, which include developing and revising goals and plans for a text reading, monitoring the reading process, and adapting behavior and strategies to achieve the reading objectives (Rollins et al. 2022).

In sum, reading comprehension involves continuously updating one’s understanding of the text as new words are read (working memory), ignoring irrelevant information (inhibitory control), switching between different cognitive tasks (cognitive flexibility), and setting and tracking reading objectives (planning and monitoring).

However, research on the role of these functions in poor comprehenders and with typical readers has yielded mixed results. For example, in a study by Locascio et al. (2010), children with reading comprehension difficulties but no word recognition problems had lower scores on planning than typical readers and poor decoders; however, no significant differences were found in inhibition and verbal working memory. On the other hand, other studies with poor comprehenders have reported deficits in verbal working memory (Jerman et al. 2012; Oakhill et al. 2005) and non-verbal working memory (Zou et al. 2022), as well as in inhibition (Borella et al. 2010). In another study conducted by Cartwright et al. (2017), poor comprehenders exhibited lower cognitive flexibility than typical readers, whereas Potocki et al. (2017) found that poor comprehenders had lower working memory, planning, cognitive flexibility, and inhibition compared to typical readers.

### 1.2. Association Between Executive Functioning and Difficulties in Fluent and Accurate Reading

Executive functioning skills, particularly working memory, inhibition, and cognitive flexibility, have also been linked to fluent and accurate reading. Working memory updating may be related to decoding, as this process requires holding phonological and morphological information in memory while processing the orthographic units being read (Ober et al. 2020). Inhibitory control can also support decoding by suppressing irrelevant information or distractions that could interfere with the process, preventing readers from impulsively guessing words based on initial letters or familiar patterns, and encouraging more accurate decoding (Diamond 2013). Furthermore, when reading irregular words in non-transparent languages, readers must inhibit the tendency to overgeneralize and apply the most common letter–sound correspondences they know in order to accurately read those words (Qiu et al. 2023). Cognitive flexibility is another skill that may be involved in decoding. In children who have yet to automate decoding and predominantly use the phonological route to read words, there is a strong need to consider various possible pronunciations of letter strings and to process different combinations of familiar letter–sound pairs (Vadasy et al. 2023). Cognitive flexibility may also enable readers to switch more easily among strategies when facing decoding difficulties. For instance, when encountering an unknown word in a text, a reader might initially sound it out phonetically but then switch to a different strategy, such as using context cues, if the word is irregular. Research with poor decoders, dyslexic readers, and dysfluent readers across several orthographies has consistently shown deficits in cognitive flexibility (Cartwright et al. 2019; Engel de Abreu et al. 2014; Moura et al. 2015), verbal working memory, and inhibition (Doyle et al. 2018; Zou et al. 2022) compared to typical readers.

Another consistent deficit found in these groups is rapid automatic naming (RAN) (Dams et al. 2023; Elwér et al. 2013). RAN is a highly complex task that entails several linguistic and cognitive skills, such as phonological abilities and processing speed, which are crucial for accurate and fluent reading (Powell and Atkinson 2021; Taub and Szente 2012). Some authors have suggested that RAN might also involve executive functioning skills, such as inhibitory control (Bexkens et al. 2015; Papadopoulos et al. 2016). RAN tasks typically require rapidly naming a number of familiar items (e.g., colors or numbers) from a set (Norton and Wolf 2012), which means that several stimuli must be kept accessible in working memory, and the activations of previously named stimuli compete with the current target stimulus for response selection. Therefore, inhibiting inappropriate response activation is necessary to choose between competing response alternatives (Bexkens et al. 2015).

### 1.3. The Present Study

In summary, research has suggested that deficits in executive functioning skills may partially explain the reading difficulties that some children experience. While research findings generally agree that difficulties in reading accurately and/or fluently are associated with poor working memory, inhibition, cognitive flexibility, and related skills such as processing speed and rapid automatized naming (RAN) (but not with higher-order processes such as planning), the results regarding poor comprehension are less consistent. Furthermore, research comparing children with fluency-only and comprehension-only difficulties to those with difficulties in both areas is scarce. Although deficits in all the aforementioned executive functions are expected due to shared impairments with the other two groups, it is unclear whether the executive functioning deficits of this group differ from a simple additive combination of those observed in the other two groups of reading difficulties—that is, whether poor readers have a distinct executive profile that is more clinically impaired. Therefore, the aim of this study was to examine differences in executive functioning among these three types of reading disabilities, comparing their performance with that of children without difficulties in either skill (typical readers). Following the above-mentioned principles (and limitations) of the Active View of Reading (Duke and Cartwright 2021), two research questions were explored: (1) Are executive functions connected to students’ reading achievement, as indicated by typical readers’ higher performance on executive functioning tests compared to groups with reading disabilities? (2) Are specific executive functioning skills differentially associated with specific reading components, as reflected in distinct patterns of executive functioning deficits among dysfluent readers, poor comprehenders, and poor readers?

## 2. Materials and Methods

### 2.1. Participants

The sample comprised 91 children (30 typical readers, 29 poor readers, 8 poor comprehenders, and 24 dysfluent readers) aged 7 to 9 years (M = 8.37; SD = 0.45), who participated in a larger study aimed at investigating their response to intervention (https://doi.org/10.54499/CEECIND/00408/2018/CP1581/CT0008 (accessed on 11 October 2024)). The students with reading difficulties were identified by their teachers and then assessed for eligibility. The typical readers were randomly selected from the same classes. All were from public schools in the northern region of Portugal.

The sociodemographic and educational characteristics of the participants are detailed in Table 1. The inclusion criteria were: (a) being a speaker of European Portuguese and having been taught to read and write in this language; (b) absence of cognitive impairment as indicated by a percentile score equal to or higher than 50 in Raven’s Coloured Progressive Matrices, and (c) absence of sensory deficits or clinical conditions affecting language development. Standardized oral reading fluency and reading comprehension tests (Rodrigues et al. 2022; Santos et al. 2017) were used to determine group membership. For the typical readers group, children had to obtain a percentile score higher than 25 in both tests. For the groups with reading difficulties, a percentile score ≤ 25 in the standardized oral reading fluency test indicated dysfluent readers, a percentile score ≤ 25 in the standardized reading comprehension test indicated poor comprehenders, and a percentile score ≤ 25 in both measures indicated poor readers. The 25th percentile has been used in other studies as the criterion for determining reading difficulties (see, e.g., Kelso et al. 2022). The groups were equivalent in terms of age, F(3, 87) = 1.913, *p* = .133, and sex, χ2(3) = 4.295, *p* = .231. However, there were significant differences in terms of SES, χ2(3) = 10.294, *p* = .016, and maternal education, χ2(6) = 15.671, *p* = .016. The proportion of poor readers and dysfluent readers from low SES backgrounds and from families with mothers who have low educational levels was higher compared to the groups of typical readers and poor comprehenders.

### 2.2. Measures

#### 2.2.1. Working Memory

The Digit Span subtest from the Wechsler Intelligence Scale for Children—Third Edition (WISC-III; Wechsler 2003) was used to measure short-term and working memory. It consists of two parts: Digit Span Forward and Digit Span Backward. In the first part, the examiner reads a sequence of single-digit numbers aloud, and the child is required to immediately repeat the numbers in the same order. The sequences start short and gradually increase in length. In the second part, the examiner reads a sequence of numbers aloud in the same way, but the child must repeat the numbers in the reverse order. As with Digit Span Forward, the sequences start short and gradually increase in length. The latter task is more challenging as it requires not only short-term memory but also mental manipulation of the information, thus engaging working memory. In the validation study for the Portuguese population, reliability coefficients, measured using the split-half method and the Spearman–Brown coefficient, for this subtest ranged between 0.71 and 0.90 (Wechsler 2003). In the present study, the value of the Spearman–Brown coefficient was 0.657.

#### 2.2.2. Cognitive Flexibility

The Trail subtest—Part B of the Coimbra Neuropsychological Assessment Battery (BANC; Moura et al. 2018) was used to measure cognitive flexibility. In part B of this subtest, the child must draw a line connecting 25 circles that contain either numbers or letters, which are randomly placed on a sheet of paper. The student must alternate between numbers and letters following alphanumeric order (e.g., 1, A, 2, B, etc.). The time taken to complete the task is then converted to standardized scores. Test–retest stability coefficient for Part B of the Trail subtest was 0.528 in the validation study for the Portuguese population (Moura et al. 2018).

#### 2.2.3. Inhibition

The Color–Word Stroop Test (Esgalhado and Pereira 2012) was used to measure inhibition. The test consists of three parts: (a) Word Reading, (b) Color Naming, and (c) Color–Word Interference. In the first part, the child is shown a list of color names (“red,” “blue,” “green”) printed in black ink and is asked to read them aloud as quickly as possible. In the second part, the child is shown a series of identical elements (“XXXX”) printed in red, blue, or green ink and is asked to name the colors of the elements as quickly as possible. In the third part, the child is shown a list of color names printed in incongruent ink colors (e.g., the word “red” printed in blue ink) and is asked to name the color of the ink rather than read the word itself. This part is the actual “Stroop” task. The interference task (Color–Word Interference) requires the child to suppress the automatic tendency to read the word and instead focus on naming the ink color. The interference t score was used in this study, with higher t scores indicating better inhibition skills. In the study of the psychometric properties with Portuguese children, this test presented an internal consistency, as measured using Cronbach’s alpha of 0.87 (Esgalhado et al. 2010). For the sample of our study, the Cronbach’s alpha for the scores in the three parts of the test was 0.76.

#### 2.2.4. Planning

Planning was assessed using the Maze subtest from the WISC-III (Wechsler 2003) and the Tower subtest from the BANC (Moura et al. 2018). In the WISC-III Maze subtest, the child is presented with a series of maze puzzles of increasing difficulty, each printed on a separate page. The child is instructed to find a path from the starting point to the endpoint without crossing any lines or hitting dead ends, completing each maze as quickly and accurately as possible within a specified time limit. Performance is scored based on the number of correct solutions and the time taken to complete the mazes. Reliability coefficients for the Maze subtest in the validation study for Portugal ranged between 0.53 and 0.80 (Wechsler 2003). In the present study, the value of the Spearman–Brown coefficient for the split-half reliability was 0.680. The Tower subtest includes 14 tasks where the child must replicate models by building a tower using three balls of different colors (red, blue, and green) and three pegs (large, medium, and small). The child must position the three colored balls on the pegs in a specified number of moves, starting with one move and progressively increasing to five moves. In the validation study for the Portuguese population, the test–retest correlation was statistically significant (*r* = 0.533), and there was evidence of construct validity (Moura et al. 2018). In the present study, the split-half reliability Spearman–Brown coefficient was 0.436.

#### 2.2.5. Rapid Naming

Two of the rapid naming subtests from the BANC (Moura et al. 2018) were used: the Rapid Automatized Naming (RAN)—Number test, which involves the rapid naming of a sequence of numbers, and the Rapid Alternating Stimulus (RAS)—Colors/Shapes test, which involves the naming of shapes and colors. In each of these subtests, children are asked to quickly identify 50 visual stimuli that are randomly arranged on a card in a 10 × 5 grid. The time taken by the student to name the stimuli is then converted into a standardized score. In the validation study for the Portuguese population, the test–retest stability coefficients were 0.802 for the RAN and 0.863 for the RAS, and evidence of construct validity was found (Moura et al. 2018).

#### 2.2.6. Processing Speed

The Coding and Symbol Search subtests from the WISC-III (Wechsler 2003) were used to measure processing speed. In the Coding subtest, children are required to copy symbols paired with simple geometric shapes or numbers based on a key provided within a specific time limit. This task evaluates how quickly and accurately the child can associate symbols with numbers and transcribe them. In the Symbol Search subtest, children are presented with a series of target symbols and must determine whether these symbols appear in a search group of symbols. This task is also timed, requiring the child to quickly and accurately identify the presence or absence of the target symbols within the search group. In the validation study for the Portuguese population, test–retest coefficients ranged between 0.49 and 0.79 for the Coding subtest, and between 0.55 and 0.65 for the Symbol Search subtest (Wechsler 2003).

### 2.3. Procedures

The study received approval from the Ethics Committee of the University of Minho (CEICSH 124/2020). Necessary legal permissions were obtained from the school boards and the parents/legal guardians of the participants. Parents/legal guardians provided informed consent for their children’s participation in accordance with the Declaration of Helsinki and the Oviedo Convention. Trained psychologists administered all measures to the students, individually and in the same order, following the instructions described in the tests’ technical manuals.

### 2.4. Statistical Analyses

Data were coded and analyzed using IBM^®^ SPSS Statistics 28. The raw scores for each subtest (except the Stroop test) were converted into (age-adjusted) scaled scores with a mean of 10 and a standard deviation of 3. The interference raw scores provided by the Stroop test were converted into t scores with a mean of 50 and a standard deviation of 10. There were no missing values in the data analyzed. An exploratory analysis was performed to check the adequacy of using parametric statistics. Univariate normality was checked by inspecting the distribution plots and the absolute values for skewness and kurtosis, which were lower than |1| for all variables. Levene’s test was not significant (*p* > .05), suggesting homogeneity of variance for all variables except the Maze scores, for which the test was significant (*p* = .044). As the parametric and non-parametric tests produced similar results for the analyses involving the Maze scores, we proceeded with the parametric statistics. Pearson correlation coefficients were calculated for all scores to check the absence of multicollinearity.

Next, a multivariate analysis of variance (MANOVA) was conducted to assess the existence of differences among the groups in executive functioning. Controlling for SES did not yield different results than not controlling for it, as SES had no significant effect in the multivariate analyses. Therefore, for the sake of parsimony, we proceeded with the analyses without SES as a variable. The assumption of homogeneity of covariance matrices was checked using the Box’s test, which yielded a non-significant result (*p* > .05). Multivariate normality was assessed using the MVN package for R (Korkmaz et al. 2014). The results suggested multivariate normality, indicated by non-significant values for multivariate kurtosis (Mardia Kurtosis = −0.44, *p* = .659) and skewness (Mardia Skewness = 186.06, *p* = .125). For the MANOVA results, partial eta squared (η^2^_p_) was used to evaluate the size of significant effects, taking into account the guidelines by Cohen (1988) for the interpretation of effects as negligible (η^2^_p_ < 0.01), small (0.01 ≤ η^2^_p_ < 0.06), moderate (0.06 ≤ η^2^_p_ < 0.14), and large (η^2^_p_ ≥ 0.14). Bonferroni’s post-hoc test was used for pairwise group mean comparisons, as it is recommended to control type I error (Field 2009). The alpha value was 0.05.

MANOVA was followed by discriminant function analysis to assess whether the dimensions of executive functioning predicted group membership. Canonical correlations were used to estimate the contribution of the predictors to the functions. Only coefficients greater than 0.30 were interpreted to distinguish between the reader groups (Tabachnick and Fidell 2013). The percentage of cases correctly classified was also calculated, including cross-validation. In cross-validation, each participant is classified by the functions derived from all participants except themselves.

## 3. Results

Table 2 presents the descriptive statistics and correlations among the dimensions of executive functioning, as well as the correlations with SES for the full sample. The mean and standard deviations for each group can be consulted in Appendix A. Most of the correlations among the assessed dimensions were low to moderate, suggesting the absence of multicollinearity. SES was correlated with RAN, RAS, and cognitive flexibility, but not with the remaining variables.

Using a significance level of α = 0.05 and a power of 0.80, sensitivity power analysis performed in G*Power 3.1.9.7 suggested a minimum detectable effect of 0.18 for Pillai V, yielding a minimum effect size of *f*^2^ = 0.093 or a η^2^_p_ = 0.085 for MANOVA main effects and *f*^2^ = 0.125 or a η^2^_p_ = 0.111 for univariate results. The results of the MANOVA revealed a significant and large effect, Pillai V = 0.587, F(27, 243) = 2.211, *p* < .001, η^2^_p_ = 0.196, suggesting differences among groups in assessed dimensions. Univariate results indicated a large difference in the two rapid naming measures, as well as in cognitive flexibility, as measured using Part B of the BANC Trail subtest, and a medium-sized difference in working memory and processing speed, as measured using the WISC-III Digit Span and Symbol Search subtests, respectively (Table 3).

**Figure 1:**
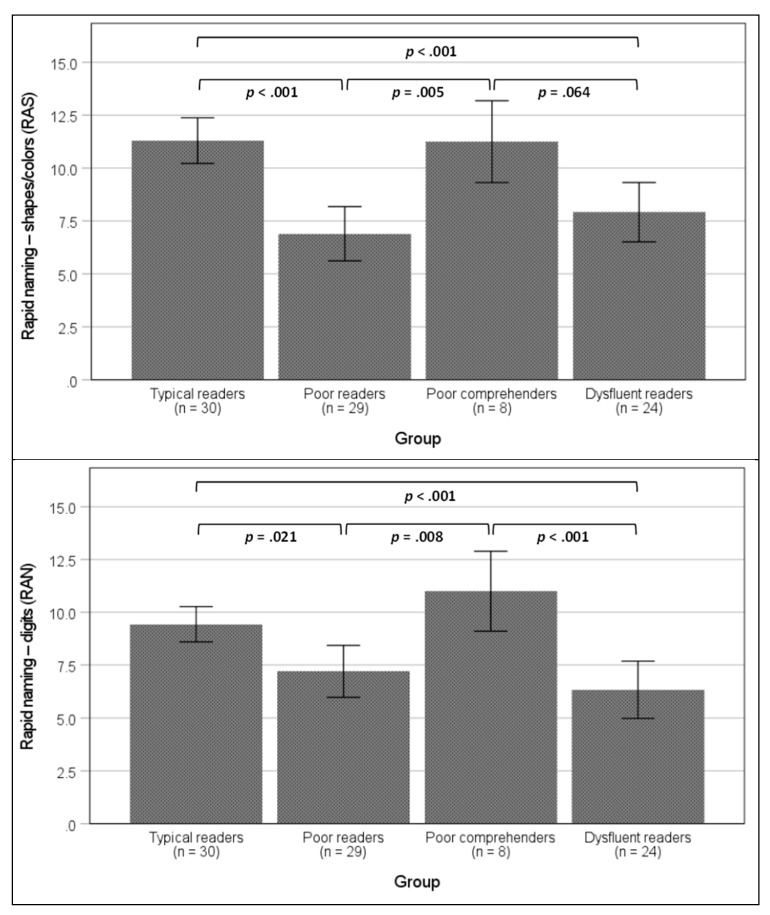
Mean differences among groups in the RAN and RAS tasks. Note: error bars represent 95% interval confidence. *p*-Values are for the Bonferroni post-hoc comparisons. Effect sizes: η^2^_p_ = 0.294 for RAS; η^2^_p_ = 0.236 for RAN.

**Figure 2:**
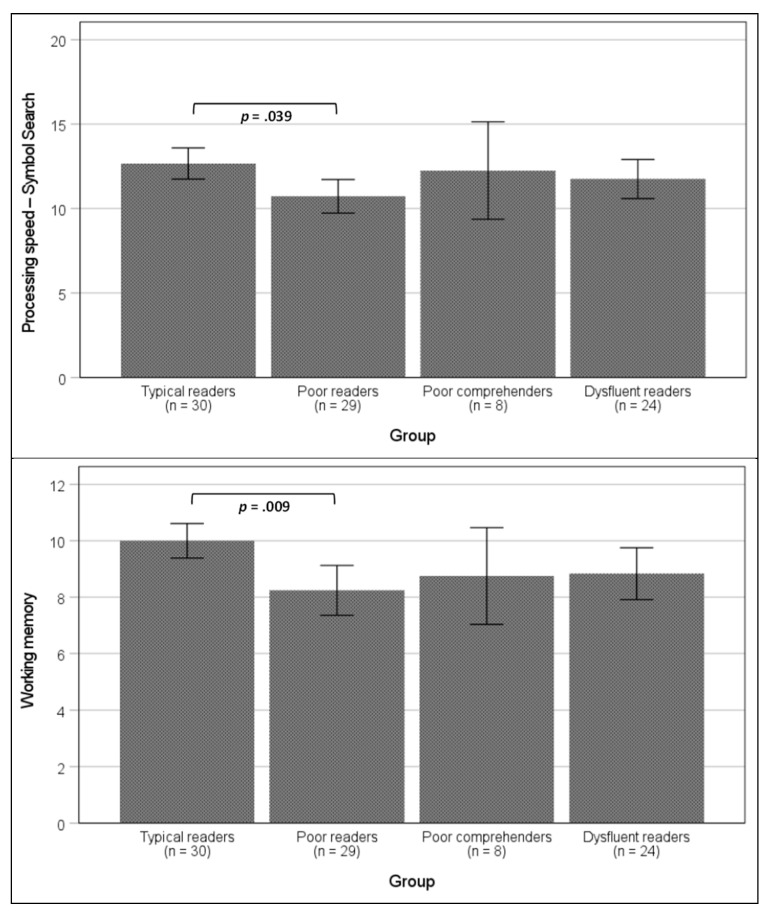
Mean differences among groups in the processing speed (symbol search) and working memory tasks. Note: error bars represent 95% interval confidence. *p*-Values are for the Bonferroni post-hoc comparisons. Effect sizes: η^2^_p_ = 0.085 for processing speed; η^2^_p_ = 0.115 for working memory.

**Figure 3:**
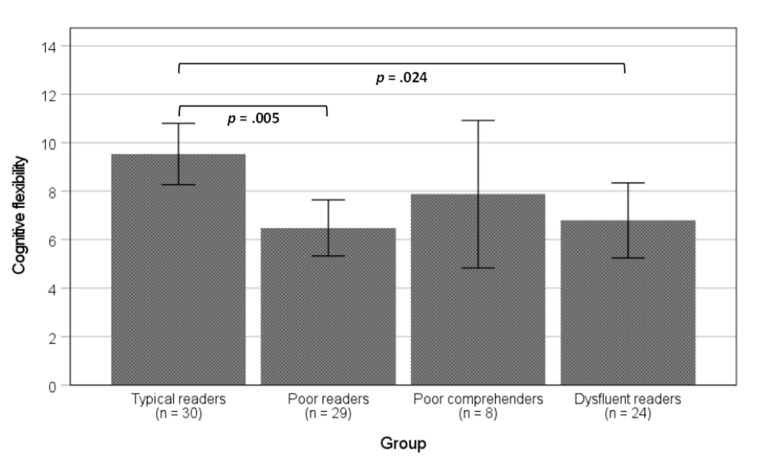
Mean differences among groups in the cognitive flexibility task. Note: error bars represent 95% interval confidence. *p*-values are for the Bonferroni post-hoc comparisons. Effect size: η^2^_p_ = 0.141.

As depicted in Figure 1, poor readers and dysfluent readers performed significantly worse than typical readers and poor comprehenders in both rapid naming tasks. In these measures, poor comprehenders had scores similar to those of typical readers. Regarding working memory and processing speed, only one pairwise difference was significant: poor readers had significantly lower scores than typical readers (Figure 2). In terms of cognitive flexibility, both poor readers and dysfluent readers performed worse than typical readers, but not significantly worse than poor comprehenders (Figure 3).

Table 4 presents the overall results of the discriminant function analysis. Three functions were calculated, with each one explaining 71.6%, 21.9%, and 6.4% of the variance, respectively. In combination, these discriminant functions significantly differentiated the readers’ groups. However, removing the first function led to the non-significance of the remaining ones, suggesting that the first function was the best to differentiate the groups (Table 4).

**Figure 4:**
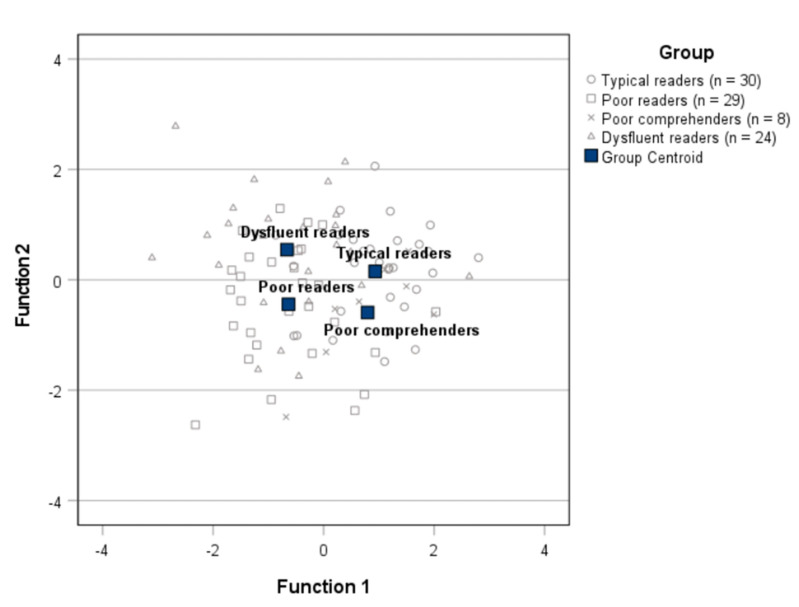
Plot of the readers groups’ centroids on the canonical discriminant functions.

Figure 4 depicts how groups are spaced along the functions according to their centroids. The inspection of this plot suggests that the first function (the significant one) separates the groups with fluency difficulties (poor readers and dysfluent readers) from the groups without fluency difficulties (typical readers and poor comprehenders). The second function discriminates between those without comprehension difficulties (dysfluent readers and typical readers) and those with (poor comprehenders and poor readers).

In the first discriminant function, the results suggested that the executive functions that best distinguished between the groups with fluency difficulties and those without were (in descending order of importance): rapid naming, cognitive flexibility, working memory, and processing speed, as measured using the WISC-III Symbol Search subtest (Table 5). Notably, performance on the Rapid Alternating Stimulus (RAS)—Colors/Shapes test had a larger loading on the first function than that on the Rapid Automatized Naming (RAN)—Number test. For the second discriminant function, the results suggested that the executive functions that best distinguished between the groups with comprehension difficulties and those without were: planning, working memory, and processing speed, again measured using the WISC-III Symbol Search subtest (Table 5). Working memory and processing speed had quite similar correlations with both functions 1 and 2.

Table 6 presents the percentage of correctly classified cases in the discriminant analysis. The results indicate that, overall, 59.3% of the cases were correctly classified (45.1% of the cross-validated grouped cases). However, the function was better at classifying the typical readers, followed by the poor readers and the dysfluent readers; however, it was not able to correctly classify the poor comprehenders (Table 6).

## 4. Discussion

The aim of this study was to examine differences in executive functioning among poor comprehenders, dysfluent readers, and poor readers, and to compare their performance with that of typical readers in order to enhance our understanding of executive profiles associated with these reading disabilities. Following the Active View of Reading (Duke and Cartwright 2021), we had two research questions related to whether typical readers in fact performed better on tests of executive functioning compared to groups with reading disabilities and whether there were distinct patterns of executive functioning deficits among dysfluent readers, poor comprehenders, and poor readers.

The results revealed lower scores in rapid naming tasks among poor readers and dysfluent readers compared to the other groups, aligning with previous research that suggests RAN deficits in children with difficulties in accurate and fluent reading (Dams et al. 2023; Elwér et al. 2013). Additionally, consistent with prior studies (Engel de Abreu et al. 2014; Moura et al. 2015), differences in cognitive flexibility were found between typical readers and the two groups with fluency difficulties, underscoring the importance of the ability to switch between information or tasks for reading. The discriminant function analysis results, which showed that performance on the task of rapid alternating among colors and shapes best distinguished readers with and without fluency difficulties, provide further insight into the importance of cognitive flexibility for fluency. Unlike the RAN task, the RAS task requires the reader to alternate between two features of the stimuli, thereby engaging cognitive flexibility.

Regarding poor comprehenders, they did not differ significantly from any of the other groups in terms of cognitive flexibility, with scores slightly above those of the groups with difficulties in reading accurately and fluently and slightly below those of typical readers. Previous studies have found an association between general cognitive flexibility and reading comprehension (e.g., Johann et al. 2020) and deficits in this executive function among poor comprehenders compared to typical readers (Cartwright et al. 2017; Potocki et al. 2017). The tendency observed in our results supports the hypothesis that cognitive flexibility plays an important role in constructing a mental model of the text, but our findings also suggest that this executive function might be more crucial for reading accurately and fluently than for reading with comprehension. The small number of poor comprehenders in our sample (and consequently the low statistical power) is an important limitation that future studies need to address. It is also important to acknowledge that we used a measure of general cognitive flexibility rather than a measure of graphophonological-semantic cognitive flexibility, which research has consistently shown to be associated with comprehension (Cartwright 2002; Cartwright et al. 2017; Gnaedinger et al. 2016). Indeed, the results of a study by Cartwright et al. (2010) involving 68 first and second-graders indicated that graphophonological-semantic cognitive flexibility was a predictor of reading comprehension, whereas general cognitive flexibility did not explain additional variance. This suggests that reading-specific cognitive flexibility is indeed a better predictor of the children’s comprehension.

No differences among the groups were found in inhibition. As previously mentioned, the role of inhibition in comprehension has yet to be consistently established, although deficits have been more consistently found in readers with decoding difficulties (Doyle et al. 2018; Zou et al. 2022). The type of measure used to measure inhibition might have contributed to the apparent inconsistency of our results with previous findings. In this study, inhibition was assessed using the interference effect in a Stroop paradigm task, which measures predominant response inhibition but does not capture other aspects of attentional control, such as resistance to distractibility—the ability to resist or resolve interference from irrelevant information during a task (Friedman and Miyake 2004). Additionally, some research on students with dyslexia has shown that, compared to typical readers, they do not exhibit general cognitive inhibition impairment; rather, their inhibition deficits are specific to reading tasks, as evidenced by lower scores in tasks such as reading sentences, where an expected word is replaced by an orthographic neighbor (Van Reybroeck and De Rom 2020).

Regarding working memory and processing speed, the findings of this study suggest mean differences between poor and typical readers. Thus, poor readers appear to have a slightly more impaired profile in terms of executive functioning than their peers who experience difficulties only in reading accurately and fluently or reading comprehension. However, the results of discriminant function analyses indicate that working memory and processing speed contribute almost equally to distinguishing students with fluency difficulties from those without, as well as those with comprehension difficulties from those without. This result is consistent with studies that have found working memory deficits in both poor comprehenders (Jerman et al. 2012; Oakhill et al. 2005) and readers with difficulties in reading accurately and fluently (Doyle et al. 2018; Zou et al. 2022).

The results of discriminant function analyses also suggest that planning is the skill that best distinguishes students with and without comprehension difficulties. The important role of planning in comprehension has been established in studies such as the one conducted by Nouwens et al. (2021), which found that working memory and planning contributed directly to reading comprehension, whereas inhibition only contributed indirectly, via decoding.

Overall, the findings indicate that the set of executive functions considered in this study is better at classifying students with poor fluency skills than those with comprehension difficulties. On the one side, this result might be partially due to the low number of poor comprehenders in the sample. On the other side, it is important to consider the possibility that other skills might be more effective in classifying poor comprehenders. Indeed, several studies have suggested that linguistic skills, such as listening comprehension or vocabulary, are the main predictors of reading comprehension, particularly after the first years of schooling when decoding is already mastered (Dolean et al. 2021; Feng et al. 2023).

As a conclusion, and taking into account our first research question, the findings of our study support the assumption of the Active View of Reading (Duke and Cartwright 2021) in terms of the involvement of executive functions in both fluency and comprehension, as typical readers scored higher than the groups with reading disabilities on a set of them. Furthermore, the results of this study revealed that there seems to be a specific pattern of deficits for each type of reading disability that cannot be explained by the architecture of this theoretical model. Another finding was that the intercorrelations among the measured executive functions were generally low to moderate. This is consistent with previous research; for example, Potocki et al. (2017) reported correlations among planning, working memory, cognitive flexibility, and inhibition ranging from −0.25 to 0.03, with the exception of the correlation between planning and working memory, which reached 0.45. Similarly, Engel de Abreu et al. (2014) found correlations among working memory (digit span), cognitive flexibility, and inhibition ranging from −0.07 to 0.31. There has been a long-standing debate about the ‘elusive nature’ or ‘task-impurity problem’ in executive functioning, which refers to the difficulty in isolating and measuring specific executive functions due to the inherent complexity of cognitive tasks that often involve multiple functions. However, studies using factor analysis have provided evidence for the existence of related but distinct factors when analyzing sets of scores across different executive functioning tasks (Himi et al. 2021; Laureys et al. 2022). Thus, the low correlations among tasks measuring the four executive functions may suggest that these tasks indeed tap into distinct executive functions. However, we must also emphasize that most of the measures used exhibit poor reliability. Low reliability is a common issue in widely used executive function measures (Burgoyne et al. 2023; Löffler et al. 2024). These measures often produce low between-subject variability, which not only leads to low reliability but also undermines correlations with other variables (Hedge et al. 2018).

Another limitation of this study is the low number of poor comprehenders in the sample. Consequently, the findings regarding this group must be interpreted with caution and future studies should include a larger number of participants with this type of reading difficulties. The method of identification we used—teacher referral—may have contributed to a high number of cases going undetected, as children with these difficulties are often overlooked in schools (Nation 2004). Implementing universal screening, followed by the administration of standardized reading tests, might lead to the identification of a greater number of poor comprehenders. Additionally, incorporating other measures of executive functioning, including the possibility of studying these relationships at the construct level, would provide a better understanding of executive profiles associated with these reading disabilities. We should also mention that the tests we used assess domain-general functions rather than domain-specific functions, which should be considered in future studies. Another limitation is the lack of equivalence among the groups in terms of SES and maternal education. Although this equivalence was not achieved, the imbalance might actually reflect the characteristics of children with reading disabilities, as several studies have shown that children from low SES backgrounds are more likely to experience reading difficulties (Buckingham et al. 2014; Cadime et al. 2021; Noble et al. 2006). We should, however, note that controlling for this imbalance statistically did not change the study findings. We must also acknowledge that the small sample size resulted in reduced statistical power, limiting the detection of only medium to large effects. However, previous research has reported medium to large differences in cognitive flexibility, processing speed, updating of working memory, and inhibition between typical readers and students with deficits in accurate and fast reading (Doyle et al. 2018; Moura et al. 2015; Zou et al. 2022), as well as in working memory, cognitive flexibility, planning and inhibition between typical readers and students with specific comprehension deficits (Borella et al. 2010; Potocki et al. 2017; Zou et al. 2022). Therefore, based on the effect sizes reported in previous research, the statistical power of our study was sufficient to detect the expected medium to large differences.

Another issue is the cutoff point (25th percentile) that we used to signalize students with reading disabilities. This cutoff value has been used in previous research (e.g., Kelso et al. 2022) but is quite liberal, as sometimes stricter cutoff values, such as the 15th percentile, have been used in some studies (e.g., Wise et al. 2008). Using more liberal cutoff values generally leads to a higher sensitivity but lower specificity, and thus more false positives may occur. Therefore, the overall accuracy of the discriminant analysis might have been affected by this choice, because when the cutoff value is too liberal, the increase in false positives may outweigh the benefit of correctly identifying true positives, thus leading to a decrease in model accuracy. Future studies should explore how different cutoff values perform in this type of analysis.

Despite these limitations, the findings of this study provide new evidence on executive functioning patterns across different types of reading disabilities in a language with intermediate orthographic depth, such as European Portuguese. Considering these findings is crucial for effective assessment and intervention with these students. On the one side, identifying the different executive functioning deficits of each group can help refine diagnostic tools and assessments, instead of a one-size-fits-all approach, which typically involves the administration of extensive and time-consuming batteries (for a review of existing batteries, see Henry and Bettenay 2010), and such assessments can be streamlined and tailored to identify students’ sources of difficulties. On the other side, recognizing different deficit profiles can help educators rethink the development of intervention programs in order to address the specific needs of each subgroup.

## Figures and Tables

**Table 1:** Major sociodemographic and educational characteristics of the sample (N = 91).

	*n*	Additional Educational Support	Age	Sex		Low SES	Maternal Education		
		*n* (%)	M (SD)	Male *n* (%)	Female *n* (%)	*n* (%)	Grade 1–9 *n* (%)	Grade 10–12 *n* (%)	Higher Education *n* (%)
Dysfluent readers	24	5 (20.8)	8.30 (0.43)	15 (62.5)	9 (37.5)	8 (33.3)	14 (58.3)	3 (12.5)	7 (29.2)
Poor comprehenders	8	1 (12.5)	8.33 (0.46)	6 (75.0)	2 (25.0)	1 (12.5)	1 (12.5)	4 (50.0)	3 (37.5)
Poor readers	29	7 (24.1)	8.29 (0.56)	12 (41.4)	17 (58.6)	13 (44.8)	11 (37.9)	12 (41.4)	6 (20.7)
Typical readers	30	2 (6.7)	8.53 (0.27)	18 (60.0)	12 (40.0)	3 (10.0)	5 (17.2)	10 (34.5)	14 (48.3)

Note: M = mean; SD = standard deviation; SES = socioeconomic status. In this study, low socioeconomic status (SES) was defined as eligibility for school-provided social support for students from disadvantaged backgrounds, which includes access to free or reduced-price meals, as well as assistance with acquiring school materials.

**Table 2:** Descriptive statistics and correlations among the assessed executive functioning dimensions and socioeconomic status (SES).

Executive Functioning Dimensions	M (SD)	1.	2.	3.	4.	5.	6.	7.	8.	9.	Low SES
1. Rapid Alternating Stimulus—Shapes/colors	9.00 (3.66)	1	0.559 ***	0.223 *	0.355 ***	0.301 **	0.209 *	0.315 **	0.424 ***	−0.020	−0.297 **
2. Rapid Automatized Naming—Digits	8.04 (3.21)		1	0.295 **	0.356 ***	0.125	0.013	0.084	0.206	−0.274 **	−0.255 *
3. Processing Speed—Coding	9.77 (3.10)			1	0.462 ***	0.088	0.043	0.279 **	0.232 *	0.008	−0.122
4. Processing Speed—Symbol Search	11.77 (2.75)				1	0.320 **	0.115	0.416 ***	0.396 ***	−0.080	−0.128
5. Planning—Maze	11.04 (2.77)					1	0.194	0.123	0.057	−0.194	−0.073
6. Planning—Tower	10.32 (3.06)						1	0.200	−0.008	0.187	0.065
7. Working Memory	9.02 (2.15)							1	0.352 ***	0.073	−0.098
8. Cognitive Flexibility	7.69 (3.60)								1	0.089	−0.271 **
9. Inhibition	50.44 (4.76)									1	−0.047

Note: M = mean; SD = standard deviation; SES = socioeconomic status. SES was coded as 0 = No; 1 = Yes. *** *p* < .001; ** *p* < .01; * *p* < .05.

**Table 3:** MANOVA univariate results.

Executive Functioning Dimensions	F(3, 87)	*p*	η^2^_p_
Rapid Alternating Stimulus—Shapes/colors	12.102	<.001	0.294
Rapid Automatized Naming—Digits	8.951	<.001	0.236
Processing speed—Coding	1.038	.380	0.035
Processing speed—Symbol Search	2.679	.052	0.085
Planning—Maze	1.762	.160	0.057
Planning—Tower	2.002	.120	0.065
Working Memory	3.766	.014	0.115
Cognitive Fexibility	4.773	.004	0.141
Inhibition	0.878	.456	0.029

**Table 4:** Results for the discriminant function analysis.

Function	Eigenvalue	% Variance	Canonical *R*^2^	Wilk’s Lambda	*p*
1	0.615	71.6	0.38		
2	0.188	21.9	0.16		
3	0.055	6.4	0.05		
1 through 3				0.494	<.001
2 through 3				0.798	.274
Only 3				0.948	.723

**Table 5:** Correlations of predictors (executive functions) with the discriminant functions.

Executive Functioning Dimensions	Discriminant Functions		
	1	2	3
Rapid Alternating Stimulus—Shapes/colors	**0.805**	0.248	−0.363
Rapid Automatized Naming—Digits	**0.658**	−0.414	−0.431
Cognitive Flexibility	**0.494**	0.204	**0.350**
Planning—Tower	−0.044	**0.529**	−0.524
Working Memory	**0.391**	**0.396**	**0.335**
Planning—Maze	0.241	**0.363**	0.061
Processing Speed—Symbol Search	**0.333**	**0.342**	−0.189
Inhibition	−0.146	0.282	0.198
Processing Speed—Coding	0.202	−0.234	−0.092

Note: Correlations between each variable and any discriminant function larger than 0.30 are in bold.

**Table 6:** Predicted group membership of the readers in each group (in percentage).

Group	Predicted Group Membership			
	Typical Readers	Poor Readers	Poor Comprehenders	Dysfluent Readers
Original				
Typical readers	**73.3**	13.3	3.3	10.0
Poor readers	6.9	**65.5**	3.4	24.1
Poor comprehenders	62.5	37.5	**0.0**	0.0
Dysfluent readers	20.8	25.0	0.0	**54.2**
Cross-validated				
Typical readers	**66.7**	16.7	3.3	13.3
Poor readers	6.9	**44.8**	10.3	37.9
Poor comprehenders	62.5	37.5	**0.0**	0.0
Dysfluent readers	25.0	37.5	4.2	**33.3**

Note: Percentage of cases correctly classified in each group are in bold.

## Data Availability

The raw data supporting the conclusions of this article will be made available by the authors upon request.

## Appendix A

**Table A1:** Means and standard deviations for each group.

Executive Functioning Dimensions	Typical Readers (*n* = 30)		Poor Readers (*n* = 29)		Poor Comprehenders (*n* = 8)		Dysfluent Readers (*n* = 24)	
	M	SD	M	SD	M	SD	M	SD
Rapid Alternating Stimulus—Shapes/colors	11.30	2.89	6.90	3.37	11.25	2.31	7.92	3.32
Rapid Automatized Naming—Digits	9.43	2.24	7.21	3.22	11.00	2.27	6.33	3.20
Processing speed—Coding	10.20	2.34	9.66	2.89	10.88	4.58	9.00	3.59
Processing speed—Symbol Search	12.67	2.47	10.72	2.62	12.25	3.45	11.75	2.74
Planning—Maze	11.83	3.35	10.21	2.02	10.88	2.30	11.13	2.72
Planning—Tower	10.20	3.01	9.48	3.36	10.25	2.31	11.50	2.72
Working Memory	10.00	1.64	8.24	2.32	8.75	2.05	8.83	2.18
Cognitive Flexibility	9.53	3.39	6.48	3.04	7.88	3.64	6.79	3.67
Inhibition	50.13	4.45	50.41	5.67	48.50	2.98	51.50	4.38

Note: M = mean; SD = standard deviation.
